# Motor Coordination in Schoolchildren Aged 6 to 11 in Northwestern Spain. A Cross-Sectional Study Based on Age and BMI

**DOI:** 10.3390/children12070814

**Published:** 2025-06-21

**Authors:** Ricardo Fernández-Vázquez, Rubén Navarro-Patón, Martín Barcala-Furelos, Javier Cachón-Zagalaz, Marcos Mecías-Calvo

**Affiliations:** 1 Faculty of Humanities and Educational Sciences, University of Jaén, 23071 Jaén, Spain; rfv00005@red.ujaen.es (R.F.-V.); jcachon@ujaen.es (J.C.-Z.); 2 Facultade de Formación do Profesorado (Campus Terra), Universidade de Santiago de Compostela, 27001 Lugo, Spain; marcos.mecias@usc.es; 3 Research Group on Motor Skills, Physical Education, and Health, Universidade de Santiago de Compostela, 27001 Lugo, Spain; martin.barcala@usc.es; 4 Faculty of Education Sciences, Universidade de Santiago de Compostela, 15782 Santiago de Compostela, Spain

**Keywords:** primary education, schoolchildren, locomotor coordination, object coordination, overweight, obesity

## Abstract

**Background/Objectives:** A large percentage of schoolchildren are children with overweight or obese, and weight gain over time increases the risk of poor health later in life. Motor coordination may be a protective factor, enabling young people to participate in healthy physical activities. However, it is unknown when the relationship between motor coordination and weight first emerges, nor whether it is evident across the body mass index (BMI) spectrum. The aim of this study was to explore what happens with coordination skills [i.e., locomotor coordination (LC), visuomotor coordination (VC), foot object control coordination (FOCC), hand object control coordination (HOCC), global motor coordination (GMC)] applying the 3JS battery, according to age (6 to 11 years) and BMI (normal weight, overweight or obesity) in a sample of 688 Primary Education participants (8.71 ± 1.66 years; 48.8% boys) from Galicia (Spain). **Methods**: To analyze the differences in the variables of the 3JS battery between age (6–11 years) and BMI (normal weight vs. overweight vs. obesity), they were evaluated using a multivariate analysis of covariance (MANCOVA), introducing the sex category (boy-girl) as a covariate to avoid possible confounding factors. **Results:** After the application of the 3JS Battery, statistically significant differences were observed depending on age [LC (*p* < 0.001); VC (*p* < 0.001); FOCC (*p* < 0.001); HOCC (*p* < 0.001); GMC (*p* < 0.001)] and BMI [LC (*p* < 0.001); VC (*p* = 0.008); HOCC (*p* < 0.001); GMC (*p* < 0.001)]. No statistically significant differences were found in the interactions between age and BMI (*p* > 0.05). **Conclusions**: Locomotor coordination increases progressively with age, both globally and in each of the manifestations, and this is greater the older the schoolchildren are. Schoolchildren with normal weight compared to schoolchildren with overweight or obesity have better global motor coordination, locomotor coordination, global control of objects, and control with the hand.

## 1. Introduction

Currently, children do not meet the minimum physical activity recommendations required for an active and healthy lifestyle [1,2]. Weight gain over time increases the risk of poor health in later life [3], leading to increased overweight and obesity [4]. Childhood overweight and/or obesity can interfere with postural control, motor performance, and motor coordination [5]. This problem worsens during childhood, a stage marked by a growing trend toward a sedentary lifestyle [6], exacerbated by an increase in passive leisure time and excessive use of technologies at early ages [7]. This is compounded by physical inactivity, exacerbated by the lockdown caused by the global SARS-CoV-2 (COVID-19) pandemic [8]. This situation is particularly acute during the transition from childhood to early adolescence, a phase in which the prevalence of obesity is significant [9] and tends to increase with age [10].

Coordination is especially important for proper human development, both physical and cognitive [11]. Motor coordination refers to the neurological organization necessary to control skeletal muscles and direct the muscular movements required to perform an activity with efficiency and precision. It is essential for performing everyday tasks such as walking, running, jumping, throwing, exercising, or playing any sport [12]. Motor competence, on the other hand, can be understood as the ability to perform different motor actions, which includes mastery of physical skills and movement patterns that enable participation in various physical activities [13].

Coordination skills evolve throughout life and can be optimized through consistent motor practice [12]. Scientific evidence indicates that the optimal age for acquiring coordination processes is between 6 and 11 years [14]. Within this age range, older schoolchildren exhibit higher levels of coordination, although this increase is not uniform [15,16] since schoolchildren show lower levels of locomotor coordination and higher levels of object control, especially with the hand, compared to the foot, except in 6-year-old schoolchildren, where coordination with the foot presents better results than coordination with the hand [17].

Scientific evidence shows that a high percentage of primary schoolchildren have insufficient levels of motor coordination [16,18] due to the low levels of physical activity they perform, both in their daily lives and in physical education [19]. Difficulties in learning coordination aspects are also evident [20], which leads to delays in learning motor skills [21]. Furthermore, schoolchildren with low levels of coordination tend not to participate in physical activities, as they may find them more difficult and may opt for other types of sedentary activities such as watching television or playing video games [5]. There is a relationship between gross motor coordination and the level of adherence to physical activity [22].

On the other hand, aspects that can improve schoolchildren’s motor coordination levels include more time dedicated to motor skills, physical and sports activity, and extracurricular activities [14]. Physically active schoolchildren who participate in extracurricular sports activities tend to have better coordination levels and a lower Body Mass Index (BMI) compared to schoolchildren who only participate in physical education and sports activities [23]. Therefore, the importance of developing coordination skills transcends the school setting, making participation in extracurricular sports activities essential [24]. Furthermore, schoolchildren with a lower BMI show better motor coordination results compared to those with a high BMI [23]. Thus, normal-weight schoolchildren aged 6 to 11 have significantly higher overall motor coordination performance than schoolchildren with overweight or obesity [18,25]. Therefore, a lower BMI is associated with less difficulty performing motor actions [26]. However, it should be noted that the relationship between BMI and hand-foot visuomotor coordination is not always significant [25,27]. In this sense, schoolchildren with obesity sometimes perform better in object manipulation than normal-weight students [25]. In this sense, developing the coordinative aspects is a complex task since there are multiple factors that influence its development, such as age, sex, strength levels, agility [28,29], level of practice and motor competence [30], body composition [19] or other more complex variables, such as the relative effect of age [29]. Thus, simultaneously analyzing BMI and age in multiple domains of motor coordination can help provide a comprehensive understanding of the factors associated with coordination deficits in the school population [18,26].

Therefore, given the scarcity of research studying the differences between age and BMI throughout Primary Education, the objective of this study was to explore what happens with Motor Coordination [i.e., locomotor coordination (LC), object control coordination (OCC), foot object control coordination (FOCC), hand object control coordination (HOCC), global motor coordination (GMC)] as a function of age and BMI (normal weight, overweight or obesity) in a sample of Primary Education schoolchildren from Galicia (Spain). In this way, the following research questions and hypotheses were raised: (1) Are there differences in motor coordination levels (locomotor, visuomotor, object control with the foot, object control with the hand, and global motor coordination) according to age in schoolchildren aged 6 to 11 years? (2) Are there differences in motor coordination levels according to body mass index (BMI: normal weight, overweight, or obesity) in schoolchildren aged 6 to 11 years?

**Hypothesis** **1.** *Older students will present better levels of motor coordination in all dimensions assessed by the 3JS battery compared to younger students.*

**Hypothesis** **2.** *The greater the degree of overweight or obesity of the students, the lower the score on each 3JS motor test and total motor coordination.*

## 2. Materials and Methods

### 2.1. Study Design

To carry out this cross-sectional descriptive design study [31], the motor coordination dimensions that make up the 3JS battery (i.e., locomotor coordination (LC), visuomotor coordination (VC), foot object control coordination (FOCC), hand object control coordination (HOCC), and global motor coordination (GMC)) were established as dependent variables. Age (6 to 11 years) and BMI (normal weight, overweight, obese) were established as independent variables. Sex (male, female) was used as a covariate.

The research was approved by the Ethics Committee of the national platform EDUCA (code 08/2024 on 10 July 2024) under the standards established in the Declaration of Helsinki.

### 2.2. Participants

Participant selection was carried out using non-probability convenience sampling, inviting a total of 750 schoolchildren from seven public schools managed by the Xunta de Galicia (Galicia Regional Government) (Spain). Selection criteria were based on geographic proximity and accessibility to the target population. For the a priori calculation of statistical power, a reference population of 91,373 primary schoolchildren enrolled in public schools was considered, according to data from the Galician Institute of Statistics (2025). The sample size was determined using the formula for finite populations [32], guaranteeing a 95% confidence level and a 4% margin of error, which indicated a minimum requirement of 597 participants.

The inclusion criteria were: (1) presentation of signed informed consent from parents or legal guardians; (2) no physical or mental illness or difficulty that would prevent participation in the 3JS battery.

Ultimately, 648 children between the ages of 6 and 11 participated. Sixty were excluded for not giving informed consent, and 42 for not attending on the day of data collection.

All participants were classified according to their BMI following the WHO [33] [i.e., 6-year-old girls (normal weight: 12.7–17.0; overweight: 17.1–19.2; obesity: 19.3 or more); 6-year-old boys (normal weight: 13.0–16.8; overweight: 16.9–18.5; obesity: 18.6 or more)].

### 2.3. Tools

Sociodemographic data, including age and sex, were collected using a specific questionnaire. Weight (kg) and height (m) were recorded to calculate BMI using the formula: weight (kg)/height (m)^2^. Weight measurement (to the nearest 0.1 kg) was performed using a TANITA DC-360 high-precision scale (Tanita, Tokyo, Japan), and height (to the nearest 0.1 cm) was measured using a TANITA Leicester HR-001 stadiometer (Tanita, Tokyo, Japan). In both tests, the students were barefoot and wearing sportswear.

The 3JS battery [34] was used for motor coordination. This battery assesses the development of global motor coordination, general dynamic coordination, and visual-motor coordination through a qualitative procedure of observation and objective evaluation of the execution of the skill developed in each task (vertical jump, turn on the longitudinal axis, slalom, precision throw, precision shot, bounce, and driving). This test is reliable for identifying changes in motor coordination in children aged 6 to 11 years, with high internal consistency (Cronbach’s alpha = 0.827), a temporal stability of 0.99, and an interobserver agreement of 0.95 [35]. This battery allows one to know the motor coordination in two specific dimensions through 7 standardized tests: (1) Locomotor Coordination (3–12): calculated through the sum of the scores obtained in the tests of vertical jump (1–4), turn on the longitudinal axis (1–4) and slalom race (1–4), (2) Total Visuomotor Coordination (4–16): Calculated through the sum of the scores obtained in the coordination of object control with the hand (2–8), Precision throw (1–4) + Rebound (1–4), and coordination of object control with the foot (2–8), Precision hit (1–4) + Impulse (1–4). Finally, total motor coordination (7–28) can be calculated by adding the 7 tests (i.e., vertical jump, turn on the longitudinal axis, slalom race, precision throw, precision hit, bounce, and impulse) [34]. In this sense, a high score on the different items in this battery has a positive meaning (greater ability to perform it), so the higher the score, the greater the motor coordination.

### 2.4. Procedures

First, the school administration and, subsequently, the physical education teachers were contacted by telephone to explain the study objectives. Second, after the study had been approved by the school administration and teachers, parents and/or legal guardians were informed and asked to sign informed consent for their children’s participation in the research. Sociodemographic data (age, sex, weight, and height) were recorded, and data from the 3JS battery were collected.

**Figure 1:**
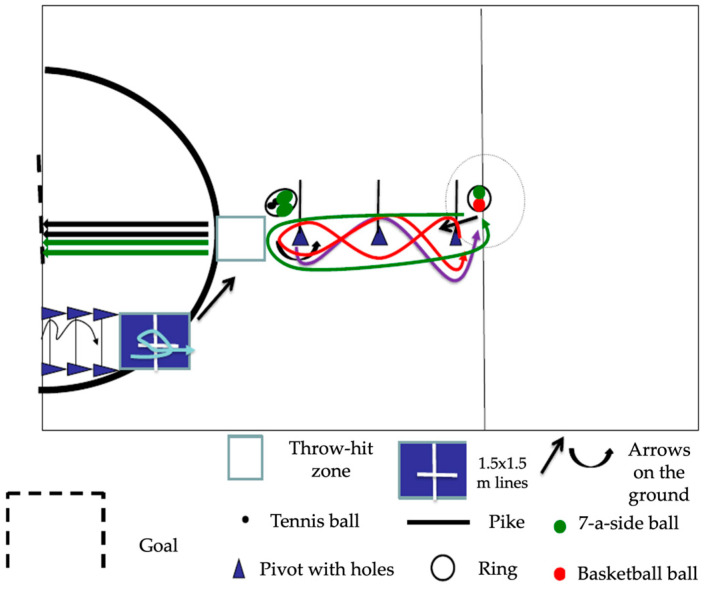
Arrangement of the material for testing the 3JS battery [35].

For data collection with the 3JS battery, the application guidelines reported by Cenizo et al. [34] were followed. A route with 7 tasks is carried out consecutively and without rest (Figure 1; 1st Vertical jump with both feet together above the pikes; 2nd Jumping upwards and turning on the longitudinal axis; 3rd Throwing two balls at a goal post from a distance without leaving the square; 4th Hitting two balls at a goal post from a distance without leaving the square; 5th Slalom running; 6th Dribbling a basketball back and forth, overcoming a simple slalom and changing direction around a pivot; 7th Driving a ball back and forth with the foot, overcoming a simple slalom and changing direction around a pivot). Performance in each of the seven tests is scored between 1 and 4 points, with 1 indicating the most immature development and 4 indicating optimal performance, following the evaluation criteria for the different tasks [35]. The materials needed to set up the circuit are 9 cones with holes, 6 cleats, 2 tennis balls, 3 soccer balls, 1 basketball ball, and 1 mat. Their distribution can be seen in Figure 1.

Standardized material was used, and the evaluators (previously trained in the use of the battery) evaluated the schoolchildren individually using the following steps: (1) Description of the tasks, (2) Practice by the schoolchildren, (3) Execution of the test following the previous instructions (no instructions were given during the test).

The 3JS battery was administered in the sports halls of the participating schools, following these instructions:

The testing area should be an open-air or covered space, 10 × 20 m, on a smooth, even surface. A half-size handball court is recommended (Figure 1):

(1) Measure 3.60 m from the goal post in the direction of the corner kick. Place the first hurdle, two cones, and a corner post. The corner post will be placed at a height of approximately 20 cm. The second hurdle will be placed 0.5 m from this first hurdle, and the third hurdle 0.5 m from it. Similarly, 0.5 m from the third hurdle, a 2 × 1 m high-density mat of a different color than the pavement will be placed for task 2. In the center of the mat, a 1 × 1 m cross will be marked with insulating tape or chalk. A visible arrow will then be marked on the ground, indicating the direction to follow for task 3.
(2) A square measuring 1.5 × 1.5 m on each side will be marked out 6 m from the end line of the field, taking as its vertex the perpendicular to the center of the right post of the 3 × 2 m handball goal. To the right (looking towards the goal), and 1 m from the center of the square, a hoop will be placed inside where approximately two yellow tennis balls and two 7-a-side football balls will be placed.
(3) One meter from the center of the goal line of the square (facing the goal), the first of the three posts with a pike inside will be placed vertically. These posts will be placed 9 m from the initial goal line; the second, 13.5 m; and the third, 18 m.
(4) At 1.5 m from the last post, another hoop similar to the previous one will be placed on the ground, inside which a basketball will be placed to carry out task 6, as well as a soccer ball 7 to carry out task 7.

### 2.5. Statistical Analysis

Categorical variables were expressed using frequency tables, and quantitative variables were expressed as measures of central tendency (mean and standard deviation). To analyze differences in the 3JS battery variables between age (6–11 years) and BMI (normal weight vs. overweight vs. obesity), a multivariate analysis of covariance (MANCOVA) was performed, introducing sex category (boys; girls) as a covariate to avoid potential confounders. The effect size was calculated using eta squared (η^2^), and the interaction between variables was calculated using the Bonferroni statistic to determine statistical significance. All statistical analyses were carried out using SPSS software (v.28, IBM Corporation, New York, NY, USA). The level of statistical significance was set at *p* < 0.050.

## 3. Results

A total of 648 healthy primary school children from Galicia, Spain, were evaluated. This was made up of 313 (48.3%) boys and 335 (51.7%) girls, with a mean age of 8.66 (SD = 1.63). The distribution of participants by BMI was 338 (59.9%) schoolchildren with normal weight, 157 (24.2%) schoolchildren with overweight, and 103 (15.9%) schoolchildren with obesity. The distribution of participants by age was 84 (13.0%) for 6 years; 98 (15.1%) for 7 years; 104 (16.0%) for 8 years; 132 (20.4%) for 9 years; 126 (19.4%) for 10 years; and 104 (16.0%) for 11 years. Table 1 shows the overall results, by age and BMI, in each of the motor coordination dimensions that were assessed using the 3JS battery.

### 3.1. Locomotor Coordination Results

The MANCOVA results for Locomotor Coordination (LC) indicated a significant main effect of the age factor [F (5, 629) = 18.153, *p* < 0.001, η^2^ = 0.126], which increased progressively with the age of the schoolchildren. A significant main effect was also found for the BMI factor [F (2, 629) = 36.227, *p* < 0.001, η^2^ = 0.103], where schoolchildren with normal weight scored higher than schoolchildren with overweight, and schoolchildren with overweight scored higher than schoolchildren with obesity. No significant differences were observed in the interaction between age and BMI (*p* = 0.885).

### 3.2. Visuomotor Coordination Results

The MANCOVA results for visuomotor coordination (VC) indicated a significant main effect of age [F (5, 629) = 34.666, *p* < 0.001, η^2^ = 0.216], which increased progressively with the age of the schoolchildren. A significant main effect was also observed for BMI [F (2, 629) = 5.385, *p* = 0.005, η^2^ = 0.017], where schoolchildren with normal weight scored higher than schoolchildren with obesity (*p* = 0.004). No significant differences were observed in the interaction between age and BMI (*p* = 0.451).

#### 3.2.1. Foot Object Control Coordination Results

The MANCOVA results for Foot Object Control Coordination (FOCC) indicated a significant main effect of age [F (5, 629) = 34.666, *p* < 0.001, η^2^ = 0.128], which increased progressively with the age of the schoolchildren. No significant main effect was observed for BMI (*p* = 0.155) or for the interaction between age and BMI (*p* = 0.495).

#### 3.2.2. Hand Object Control Coordination Results

The results of the MANCOVA on Hand Object Control Coordination (HOCC) indicated a significant main effect of age [F (5, 629) = 39.104, *p* < 0.001, η^2^ = 0.237], which increased progressively with the age of the schoolchildren. A significant main effect was also observed for BMI [F (2, 629) = 8.014, *p* < 0.001, η^2^ = 0.025], where schoolchildren with normal weight scored higher than schoolchildren with obesity (*p* = 0.001). No significant differences were observed in the interaction between age and BMI (*p* = 0.246).

### 3.3. Global Motor Coordination Results

The MANCOVA results for Global Motor Coordination (GMC) indicated a significant main effect of age [F (5, 629) = 34.561, *p* < 0.001, η^2^ = 0.216], which increased progressively with the age of the schoolchildren. A significant main effect was also observed for the BMI factor [F (2, 629) = 19.781, *p* < 0.001, η^2^ = 0.059], where schoolchildren with normal weight scored higher than schoolchildren with overweight and schoolchildren with overweight scored higher compared to schoolchildren with obesity. No significant differences were observed in the interaction between age and BMI (*p* = 0.628).

## 4. Discussion

The aim of this study was to explore motor coordination [i.e., locomotor coordination (LC); visuomotor coordination (VC); foot object control coordination (FOCC); hand object control coordination (HOCC); global motor coordination (GMC)] and BMI (normal weight, overweight, or obesity) in a sample of primary school children from Galicia, Spain.

The trend towards a sedentary lifestyle is known to be increasing [6], and physical inactivity was further exacerbated by the global SARS-CoV-2 (COVID) pandemic, increasing rates of overweight and obesity [8]. In this sense, of the 648 primary schoolchildren studied, 338 were children with normotypical BMI, 157 were children with overweight, and 103 were children with obesity. Comparing our data with the study by Rodríguez Cayetano et al. [36] with 671 primary school children, higher rates of schoolchildren with normal weight (438) and lower rates of schoolchildren with overweight (116) and obesity (47) were observed compared to the present study. Therefore, it can be stated that the trend towards overweight and obesity continues to increase.

### 4.1. Locomotor Coordination

The results of this study indicate that locomotor coordination considerably improves with age [37]. Furthermore, in parallel with the development of coordination, de Chaves et al. [37] also identified an increase in coordination deficits with age. Therefore, scientific evidence establishes a direct association between locomotor coordination and the level of physical activity practiced [22]. Therefore, to understand the causes that lead to coordination deficits in Primary Education ages, it is necessary to take into account the low levels of physical activity among the child population [1,2].

In this sense, the results of the present study corroborate the existence of a significant relationship between BMI and the level of locomotor coordination since schoolchildren with obesity obtain worse results in locomotor coordination than schoolchildren with overweight and, in turn, schoolchildren with overweight also show worse results than schoolchildren with normal weight. In this sense, Martínez García et al. [25] observed significant differences in all coordination variables in schoolchildren aged 6 to 9 years, with schoolchildren with normal weight showing better locomotor performance compared to schoolchildren with overweight or obesity.

Regarding the interaction of age and BMI, no significant differences were observed in the present study, which could be explained by taking into account that the main factor that improves the level of locomotor coordination by schoolchildren is the increase in motor engagement time, followed by the performance of physical and sports activity, both inside and outside the classroom [14].

### 4.2. Visuomotor Coordination

The results related to Visuomotor Coordination (VC) show that it increases progressively as the age of the schoolchildren increases [15,16], obtaining better results in VC compared to LC [16]. In this study, VC is composed of object control with the foot and object control with the hand. Therefore, after the results obtained in these two dimensions, both the results related to object control coordination with the hand (HOCC) and with the foot (FOCC) reflect that there is a significant effect of the age factor, both increasing significantly as age increases, unlike the results found by Cenizo-Benjumea et al. [16] where the results in object control with the foot are lower than those achieved with the hand, this difference increasing at older ages.

In the case of the FOCC, no significant effect was obtained in relation to BMI or in the interaction between age and BMI. This result can be explained considering that, although coordination tends to get better with age [17] and a better BMI is usually associated with better coordination indices [23], excess weight can act as a compensatory factor in certain motor areas since overweight and obese schoolchildren between 5 and 8 years old have higher levels of strength in their feet, while overweight and obese schoolchildren between 7 and 8 years old show greater stability in the lower body [38]. This null finding from the present study suggests that a higher BMI may sometimes compensate for or even bias performance on foot coordination tasks, which could explain the lack of significant differences in this area.

Significant differences in BMI were also found for object control coordination with the hand (HOCC). Schoolchildren with normal weight scored higher on the HOCC than schoolchildren with obesity. However, in other studies [25,27], no significant differences were found between schoolchildren with normal weight and schoolchildren with overweight or obesity in object control. Thus, although improved coordination in object control could influence an improvement in BMI [23], scientific evidence shows that visual-motor coordination tests are not always statistically significant in relation to the BMI factor [23,25].

### 4.3. Global Motor Coordination

The results of the present study indicate that global motor coordination (GMC) rises substantially with age, as in the study by Rosa Guillamón et al. [18], where 8-year-old schoolchildren obtain better results in global motor coordination compared to 6-year-old schoolchildren.

Regarding the BMI variable, in relation to global coordination, the present study shows that schoolchildren with obesity obtain worse results in global coordination than schoolchildren with overweight, and, in turn, schoolchildren with overweight show worse results than schoolchildren with normal weight. These results are in line with the study by Martínez García et al. [25], where schoolchildren with normal weight have better global motor coordination than schoolchildren with overweight or obesity. In this sense, the improvement in global motor coordination and its coordinative effectiveness could be associated with the practice of sport or federated physical activity of schoolchildren, with those who practice it obtaining better motor performance compared to those who do not practice sport or physical activity [18]. Thus, the present study can provide another variable that allows explaining the causes of coordination deficits, and it is the influence of the BMI itself, given that schoolchildren with a higher BMI generally have worse motor coordination [23].

Regarding the study’s limitations, it is worth highlighting its cross-sectional design, which prevents establishing causal relationships, and the lack of assessment of other factors, such as socioeconomic status and behavioral factors (e.g., extracurricular activities and screen time). Furthermore, it is worth noting that, to date, there are few studies that use the 3JS Motor Coordination Test to assess the level of general motor coordination in primary schoolchildren. In this sense, taking into account all the limitations, the results should be interpreted with caution.

## 5. Conclusions

This study confirms that age and BMI are key factors in the development of motor coordination in primary schoolchildren. While age is associated with generalized improvement in all motor areas, being a schoolchild with overweight negatively affects locomotor coordination and hand object control coordination in particular.

Regarding age, the results showed that it is a determining factor in the development of all dimensions of motor coordination assessed. Despite this, some coordination skills are more affected, such as object control with the hand and general motor coordination.

Regarding BMI, the results confirmed that it has a significant impact on motor coordination, although with variations depending on the dimension assessed. For example, schoolchildren with normal weight obtained significantly higher scores than those with overweight or obesity, especially in locomotor coordination. However, no significant differences were found in foot object control coordination (FOCC), suggesting that this skill may be less influenced by body weight than other dimensions.

The results of this study have important implications for school physical education. On the one hand, age-specific interventions are proposed that address motor coordination as it evolves with age. Furthermore, curricular reinforcement could be included for specific skills where age has a greater impact (e.g., the HOCC). Therefore, physical education teachers should adapt this content to schoolchildren’s developmental levels, progressively introducing more complex tasks. Furthermore, given that a higher BMI indicates lower motor performance, the need for strategies to combat sedentary lifestyles and promote healthy habits from an early age in schools should be reinforced (e.g., the inclusion of active playgrounds).

As future lines of research, we propose conducting longitudinal studies to analyze individual motor development and explore how some variables (extracurricular sports practice) interact with BMI, age, gender, and coordination.

## Figures and Tables

**Table 1:** 3JS test results according to age and BMI.

Age		6	7	8	9	10	11
		M ± SD	M ± SD	M ± SD	M ± SD	M ± SD	M ± SD
**LC** (3–12)	Normal weight	7.87 ± 2.15	9.72 ± 1.69	9.90 ± 1.48	10.13 ± 1.62	10.30 ± 1.35	10.39 ± 1.49
	Overweight	6.57 ± 1.71	9.04 ± 1.75	9.13 ± 1.78	9.85 ± 1.71	9.76 ± 1.51	9.81 ± 1.57
	Obesity	6.09 ± 2.38	7.41 ± 2.06	8.70 ± 2.02	8.70 ±1.89	8.78 ± 1.57	8.76 ± 2.42
**VC** (4–16)	Normal weight	7.83 ± 2.53	9.77 ± 2.48	10.56 ± 2.53	11.47 ± 2.85	11.75 ± 2.50	12.22 ± 2.79
	Overweight	6.71 ± 2.21	9.59 ± 2.70	10.86 ± 2.31	11.17 ± 2.59	11.71 ± 2.77	11.45 ± 2.50
	Obesity	6.00 ± 2.19	7.47 ± 2.26	10.52 ± 2.28	10.17 ± 2.76	11.78 ± 2.37	11.38 ± 3.99
**FOCC** (2–8)	Normal weight	4.00 ± 1.62	4.69 ± 1.45	5.13 ± 1.57	5.61 ± 1.54	5.73 ± 1.54	5.81 ± 1.78
	Overweight	4.00 ± 1.73	4.63 ± 1.59	5.54 ± 1.43	5.40 ± 1.49	5.65 ± 1.72	5.36 ± 1.83
	Obesity	3.88 ± 1.37	3.64 ± 1.16	5.52 ± 1.69	4.76 ± 1.60	5.67 ± 1.49	5.69 ± 2.05
**HOCC** (2–8)	Normal weight	3.83 ± 1.22	5.08 ± 1.29	5.43 ± 1.32	5.86 ± 1.51	6.01 ± 1.25	6.41 ± 1.28
	Overweight	2.71 ± 1.11	4.95 ± 1.46	5.31 ± 1.12	5.77 ± 1.28	6.05 ± 1.20	6.09 ± 1.04
	Obesity	2.90 ± 1.04	3.82 ± 1.59	5.00 ± 1.73	5.41 ± 1.62	6.10 ± 1.13	5.69 ± 2.17
**GMC** (7–28)	Normal weight	15.71 ± 4.04	19.50 ± 3.69	20.47 ± 3.42	21.61 ± 3.97	22.05 ± 3.27	22.62 ± 3.85
	Overweight	13.28 ± 3.45	18.63 ± 4.05	20.00 ± 3.50	21.02 ± 3.67	21.47 ± 3.87	21.27 ± 3.57
	Obesity	12.09 ± 2.38	14.88 ± 4.12	19.23 ± 4.99	18.88 ± 4.24	20.57 ± 3.43	20.15 ± 6.14

Note: M = Mean; SD = Standard deviation; LC = Locomotor coordination; VC = Visuomotor coordination; FOCC = Foot object control coordination; HOCC = Hand object control coordination; GMC = Global motor coordination.

## Data Availability

The data presented in this study are not available in accordance with Regulation (EU) of the European Parliament and of the Council 2016/679 of 27 April 2016 regarding the protection of natural persons with regard to the processing of personal data and the free circulation of these data (RGPD).

## Abbreviations

The following abbreviations are used in this manuscript:

LC	Locomotor coordination
VC	Visuomotor coordination
FOCC	Foot object control coordination
HOCC	Hand object control coordination
GMC	Global motor coordination
COVID-19	SARS-CoV-2
BMI	Body Mass Index
MANCOVA	Multivariate analysis of covariance
